# ^18^F-FDG PET/MRI Imaging in a Preclinical Rat Model of Cardiorenal Syndrome—An Exploratory Study

**DOI:** 10.3390/ijms232315409

**Published:** 2022-12-06

**Authors:** Dan Mihai Furcea, Laurențiu Agrigoroaie, Cosmin-T. Mihai, Ioannis Gardikiotis, Gianina Dodi, Gabriela D. Stanciu, Carmen Solcan, Sorin I. Beschea Chiriac, Mihai Marius Guțu, Cipriana Ștefănescu

**Affiliations:** 1 Department of Nuclear Medicine, Sf. Spiridon University Emergency Hospital, 700111 Iasi, Romania; 2 Advanced Research and Development Center for Experimental Medicine, Grigore T. Popa University of Medicine and Pharmacy of Iasi, 700454 Iasi, Romania; 3 Faculty of Veterinary Medicine, Ion Ionescu de la Brad University of Agricultural Sciences and Veterinary Medicine, 700490 Iasi, Romania; 4 Department of Biophysics and Medical Physics—Nuclear Medicine, Grigore T. Popa University of Medicine and Pharmacy of Iasi, 700115 Iasi, Romania

**Keywords:** cardiorenal syndrome, animals, PET, MRI, histopathology, immunohistochemistry

## Abstract

Cardiorenal syndrome (CRS) denotes the bidirectional interaction of chronic kidney disease and heart failure with an adverse prognosis but with a limited understanding of its pathogenesis. This study correlates biochemical blood markers, histopathological and immunohistochemistry features, and 2-deoxy-2-fluoro-D-glucose positron emission tomography (^18^F-FDG PET) metabolic data in low-dose doxorubicin-induced heart failure, cardiorenal syndrome, and renocardiac syndrome induced on Wistar male rats. To our knowledge, this is the first study that investigates the underlying mechanisms for CRS progression in rats using ^18^F-FDG PET. Clinical, metabolic cage monitoring, biochemistry, histopathology, and immunohistochemistry combined with PET/MRI (magnetic resonance imaging) data acquisition at distinct points in the disease progression were employed for this study in order to elucidate the available evidence of organ crosstalk between the heart and kidneys. In our CRS model, we found that chronic treatment with low-dose doxorubicin followed by acute 5/6 nephrectomy incurred the highest mortality among the study groups, while the model for renocardiac syndrome resulted in moderate-to-high mortality. ^18^F-FDG PET imaging evidenced the doxorubicin cardiotoxicity with vascular alterations, normal kidney development damage, and impaired function. Given the fact that standard clinical markers were insensitive to early renal injury, we believe that the decreasing values of the ^18^F-FDG PET-derived renal marker across the groups and, compared with their age-matched controls, along with the uniform distribution seen in healthy developing rats, could have a potential diagnostic and prognostic yield in cardiorenal syndrome.

## 1. Introduction

Cardiorenal syndrome (CRS) is an intricate clinical condition where chronic kidney disease (CKD) and heart failure (HF) coexist and evolve by system crosstalk, and the failure of one organ accelerates the progression of structural damage and failure in the other organ. When the acute or chronic malfunction of the left ventricle co-evolving with acute or chronic renal disease, the emerging clinicopathological entity is classified as CRS, and it leads to the progressive deterioration of both systems resulting in high morbidity and mortality [1]. Five subtypes of CRS have been identified [2] and are briefly described as type 1 and type 2, where acute/chronic cardiac dysfunction leads to acute kidney injury; type 3 and type 4 are where acute/chronic kidney dysfunction precedes and contributes to the development of cardiac injuries; and type 5 is defined as simultaneous heart and kidney dysfunction due to acute or chronic systemic condition.

Experimental models play a crucial role in analyzing the complex interplay between the heart and the kidney in evolving disease procession [3].

In 2015, type 4 CRS was induced by Chang et al. [4] on male Sprague Dawley rats by subtotal nephrectomy in two steps: initial kidney injury by first removing the entire right kidney and, one week later, by resecting two-thirds of the left kidney. The authors synthesized and used, for the first time, a dual-modality magneto-fluorescent nanoparticle agent to detect cardiac inflammation and monitor the therapeutic efficacy of anti-inflammatory treatment by MRI and optical imaging. The MRI, optical imaging, and histopathological results revealed essential pathophysiological changes in myocardial inflammation in the progression of type 4 CRS [4]. Chuppa et al. [5] observed the miR-21-5p expression changes throughout the development of cardiac pathology in the 5/6 nephrectomy model of type 4 CRS on male Sprague Dawley rats. According to the obtained results, miR-21-5p was recognized as a mediator of left ventricular remodeling and dysfunction through its regulation of peroxisome proliferator-activated receptor-α (PPARα). In 2018, the first renal proteomic analysis of type 2 CRS changes was made on experimental chronic HF models induced in male Wistar rats using an aortocaval fistula. The analysis identified and quantified almost 4000 proteins and established the role of intrarenal angiotensin-II signaling along with several other potential participants, such as the receptor for advanced glycation end products (RAGE), Periostin, Caveolin-1, von Willebrand factor and Galectin-3 on HF-induced kidney injury [6]. The role of Wnt/b-catenin signaling as a common pathogenic mediator of heart and kidney injury was investigated on the type 2 CRS model induced by transverse aortic constriction (TAC) on male C57BL/6 mice. At 8 weeks, after TAC, a cascade of events such as cardiomyocyte hypertrophy, inflammation, and kidney fibrosis, were present, along with impaired cardiac function, confirmed by echocardiography. The authors considered that, by targeting this pathway, both organs could be protected from progressing to CRS [7]. A similar model of CRS, as the one used in our study, was developed by 5/6 subtotal nephrectomy and doxorubicin treatment on male Sprague Dawley rats and was used to evaluate the role of early administration of empagliflozin on heart function preservation. Cell culture and in vitro studies were combined with functional cardiac ultrasound assessment, plasma creatinine levels, qualitative analysis of kidney injuries by immunohistochemical and immunofluorescent staining, and histopathological findings in order to demonstrate that empagliflozin therapy preserved heart and renal function and inhibited left ventricular remodeling through the inhibition of molecular-cellular perturbations [8].

Taking into consideration the knowledge disclosed so far by experimental studies in CRS, it appears that a consensus is building around the paradigm of increased oxidative stress, the generation of oxygen reactive species (ROS), increased inflammation, and cellular apoptosis [9]. Additionally, previous studies on doxorubicin-induced cardiotoxicity revealed severe cellular injuries, increased inflammation, oxidative stress, cellular apoptosis or fibrosis, deoxyribonucleic acid (DNA) lesions, and mitochondrial dysfunctions [10]. These cellular alterations are increasingly recognized and underlined in doxorubicin-induced nephropathy as well [11]. Cardiac dysfunction can be examined from the standpoint of a 5/6 nephrectomy model that replicates clinical cardiac uremic syndrome [12].

Currently, despite the different imaging methods for single-photon emission computerized tomography (SPECT) and MRI being established for CRS pathophysiology assessment, the ability to accurately evaluate vascular alterations has been limited. ^18^F-FDG PET is a widely accepted tool in clinical practice for myocardial viability studies in coronary syndromes [13], cardiac inflammation [14], prosthetic valve infections [15], and in guiding revascularization decisions in ischemic heart failure [16], mirrored by preclinical analysis in myocardial glucose metabolism estimations and therapeutics testing [17]. Due to the excretion route of this tracer, kidney applications are lacking in the clinical arena [18,19,20,21], and its’ main uses are restricted to preclinical studies [22,23,24,25,26,27,28]. Recently [29], PET with the specific CXCR4 ligand ^68^Ga-Pentixafor was performed after myocardial infarction in mice, and the images revealed a tight inflammatory interaction between the myocardium and the kidneys as secondarily affected organs after primary cardiac injury. In a translational approach, cardiac CXCR4-directed PET imaging also outperformed the established clinical parameters in identifying patients at increased risk for worsening renal function, which could bear potential for the image-based guidance of renoprotective strategies early and after acute myocardial infarction. 

Our aim was to study the relationships between routine biochemical blood markers, histopathological features, and ^18^F-FDG PET metabolic data in the setting of a progressive heart and kidney failure animal model. To our knowledge, this is the first study that investigates the underlying mechanisms for CRS progression in Wistar rats using ^18^F-FDG PET.

## 2. Results

### 2.1. Clinical and Biochemical Characteristics

**Figure 1:**
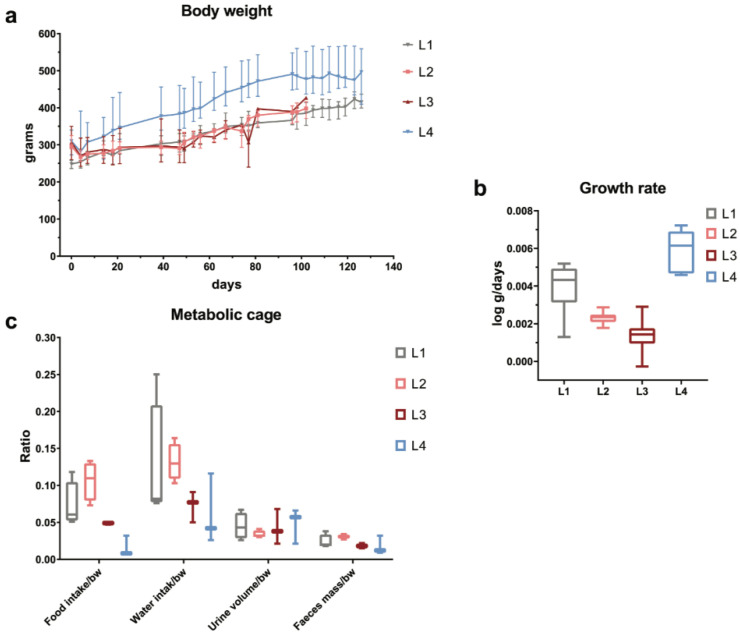
Clinical monitoring by body weight (**a**,**b**) and metabolic cage (**c**) where doxorubicin toxicity became apparent in L2 and L3 groups by reducing growth rate and influencing eating behavior, while in L4 rats an increase in growth rate despite reduced food and water intake was observed.

Over the observation period, L2 and L3 individuals displayed moderately blunted weight gain patterns with a tendency toward the reduction in growth rates, whereas L4 had an increasing pattern of weight gain, entering a plateau after the first week of the doxorubicin regimen (Figure 1a,b). Metabolic cage monitoring resulted in the reduction in food and water intake for the L3 and L4 groups, while their urinary and stool output did not differ. In contrast, L2 individuals manifested a tendency for the increased input of rat chow, while water intake was similar to the control group, albeit the latter had an increased variation (Figure 1c). 

**Figure 2:**
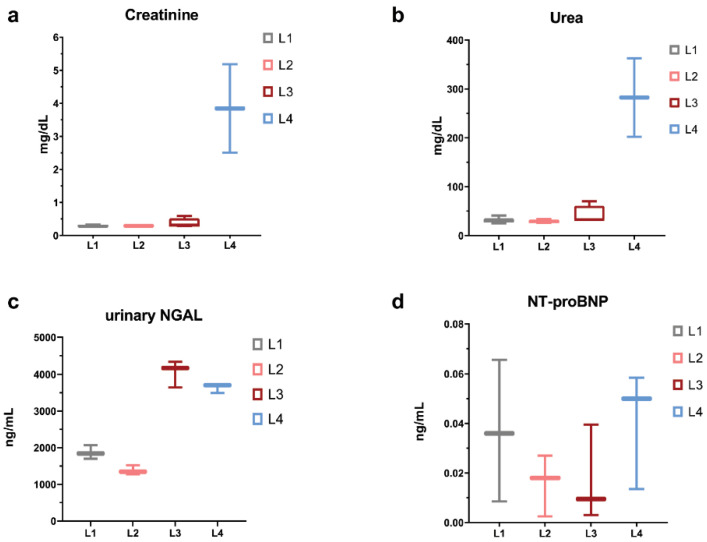
Serum biochemical parameters, as a snapshot taken at end of study, disclosing an apparent lack of doxorubicin renal (**a**,**b**) and cardiac toxicity (**d**), while the urinary tubular marker NGAL (**c**) differentiates between early and delayed doxorubicin-treated rats, as well as for renocardiac group.

Concerning serum urea and creatinine levels, we observed marked elevations only for the L4 group (Figure 2a,b). Urinary NGAL values for L2 individuals were similar to the L1 group, while L3 and L4 were several folds higher than the control (Figure 2c), whereas NT-proBNP did not show elevations across the groups (Figure 2d). Hence, it seems that the L3 group, doxorubicin-treated with delay, manifested biochemical evidence of tubular damage but no glomerular impairment, whereas L2 rats, doxorubicin-treated without delay, lacked any sign of renal injury at the moment of measurement (the end of the study). In comparison to all other groups, L4 subjects displayed clear evidence of both glomerular and tubular damage.

### 2.2. Histopathology and Immunohistochemistry Evaluation

**Figure 3:**
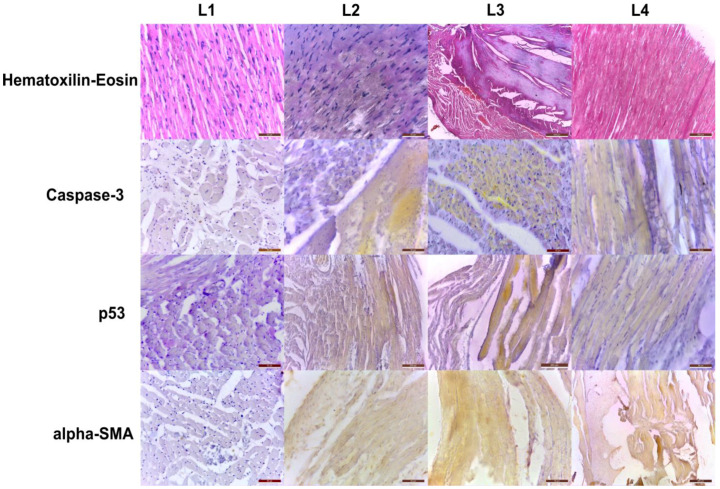
Cardiac histopathology, with representative slides for each group, demonstrates progressive lesions with focal necrosis, disorganization of myofibrils and intracytoplasmic vacuolization with close matching of immunohistochemistry markers of genotoxicity, apoptosis, and fibrosis (left panels, scale bar = 20-50-50-50 μm, middle panels, scale bar = 50-50-200-50 μm and 500-50-200-50 μm and right panels, scale bar = 200-50-50-500 μm; 10-20-100× magnification).

On the qualitative examination of stained myocardial slides, we noticed progressive alterations in the tissue structure for the L2, L3, and L4 groups, in contrast to the control group, with foci of necrosis, dissolution of intracytoplasmic myofibrils, and cardiomyocytes with intracytoplasmic vacuolization (Figure 3). Additionally, the observed histological modifications were closely followed by the immunohistochemistry markers evaluation for genotoxicity, apoptosis, and fibrosis.

**Figure 4:**
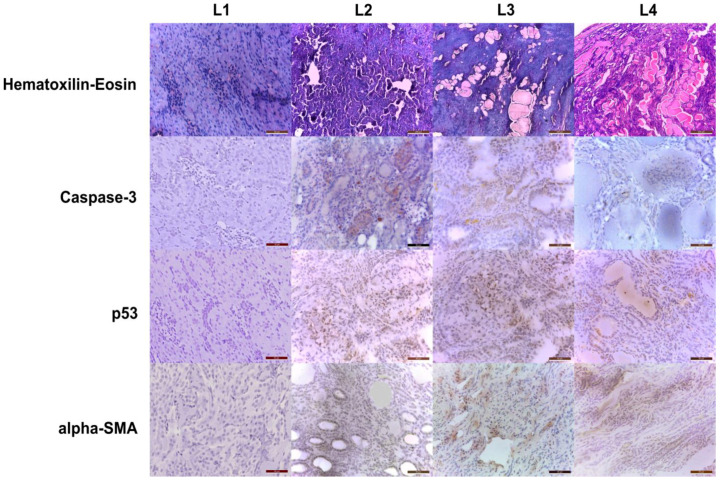
Renal histopathology slides point to progressive toxicity across groups, with increasing areas of glomerular sclerosis and tubular atrophy paralleled by an increased density of expression for genotoxic, apoptosis, and fibrosis markers (left panels, scale bar = 20-50-50-50 μm, middle panels, scale bar = 200-50-50-50 μm and 500-50-50-50 μm and right panels, scale bar = 500-50-50-50 μm; 10-20-100X magnification).

In renal specimens, group membership was better predicted by distinct profiles of the lesion involvement and extent. While the L2 group subjects displayed a few foci of glomerular and tubular sclerosis, some hyaline casts, and collecting tubule ectasia, the L3 and L4 groups presented a greater extent of glomerular sclerosis with areas of vacuolization and proteinaceous deposits (Figure 4). Additionally, the tubular system exhibited an increased magnitude of luminal effacement, hyaline casts, and connective tissue proliferation, particularly in medullary regions. Immunohistochemical staining for genotoxicity, apoptosis, and fibrosis in the affected areas demonstrated an increasing involvement in the glomeruli and tubules across the groups (Figure 4).

### 2.3. MRI Organ Volumetry

**Figure 5:**
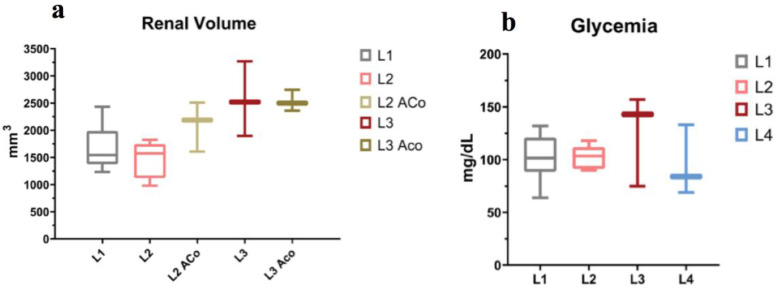
(**a**) Influence of doxorubicin on kidney volumes right after completion of the 6 weeks course (L2) and after another 6 weeks from regimen termination (L3) and their age-matched counterparts; (**b**) Pre-scan blood glucose levels within acceptable range for ^18^F-FDG PET study (<200 mg/dL), with a tendency towards the upper limit for cardiorenal rats.

A distinct observation could be made with regard to the evolution of the renal volumes throughout the study (Figure 5a). Organ measurements were conducted at the end of the study period of each group, with renal volume estimation by approximation with an ellipsoid. Compared to L1 (1590 mm^3^), measured at 14 weeks of life, the L2 group (1480 mm^3^), measured at 20 weeks (after 6 weeks of doxorubicin exposure), displayed not only a blunting in the normal growth trajectory of kidney volume but also a regression when compared to the age control rats (2101 mm^3^). However, in the case of L3 rats (measured at 26 weeks), considered delayed doxorubicin-treated, there was an apparent recovery in kidney growth, which we hypothesized was based on the increasing fibrosis and accumulation of immune cells.

### 2.4. PET Data

As a gross index of glucose homeostasis, the circulating levels of blood glucose were measured immediately before radiotracer administration when the rat was already under isoflurane anesthesia for at least 20 min. Despite several studies [30] proving isoflurane’s effect on increasing glycemic levels, in our data, only the L3 group rats displayed a tendency toward higher levels (Figure 5b).

Another aspect to be taken into account was the site of the radiotracer administration and the shape of the blood time–activity curve. During preliminary data analysis, we observed in some of the rats a persistently ascending curve of the vascular ROI without any sign of flattening by the end of the scan duration. This behavior in radiotracer distribution, in conjunction with the observations made by Tse et al. [31], led us to the supposition of a local tail depot effect, and the PET data analysis for these rats was discarded.

It must be noted, as a perspective for future studies, that a glucose tolerance test would give additional insights on glucose homeostasis since anesthesia has been shown to affect blood glucose levels.

#### 2.4.1. Static Data

**Figure 6:**
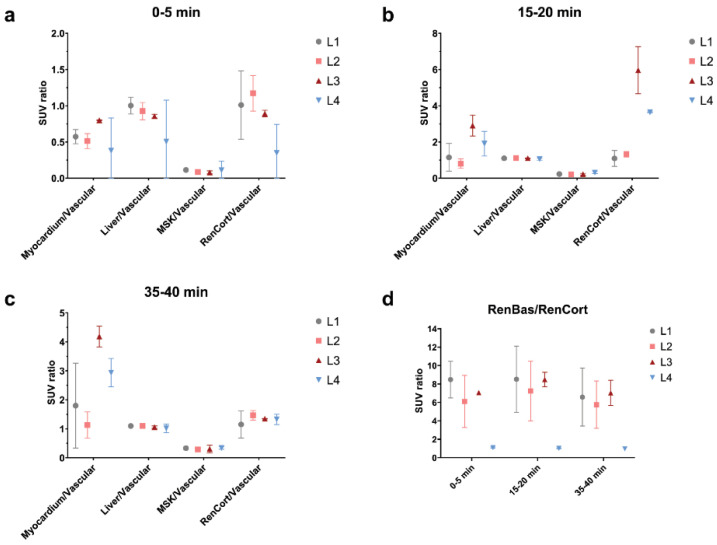
Static ^18^F-FDG PET target organ SUV normalized to blood for TP1 (**a**), TP2 (**b**), and TP3 (**c**) and also as a proxy for renal output with kidney pelvis SUV normalized to kidney cortex (**d**).

As a first step for the analysis of PET data, we chose to partition it into three static frames, corresponding to an early perfusion-dominated phase (0–5 min), an intermediate (15–20 min), and a late uptake-dominated phase (35–40 min). The blood normalized SUV values for the skeletal muscle and liver demonstrated no difference in the uptake across all time spans and groups, except for the first phase in the liver, where a tendency for a decreased distribution of ^18^F-FDG was observed (Figure 6) when compared with the other two uptake phases, intermediate and late, respectively. The relative consistency of these two PET markers can be seen as an internal control for the glucose metabolism steady state.

**Figure 7:**
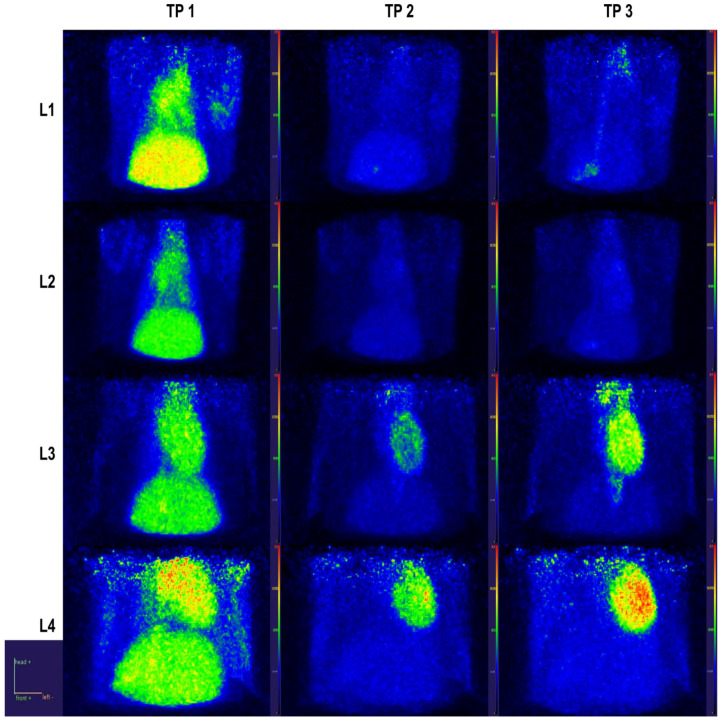
Heart and liver close-ups in parametric maximal intensity projection images with voxel-wise normalization to blood SUV, depicting different behaviors of ^18^F-FDG according to time interval and group (Note: the orange-red and yellow color represents the highest uptake regions, blue and green colors show areas of decreased metabolic activity).

Regarding target organ metabolic activity, myocardium uptake was similar for the L1 and L2 groups throughout these intervals, while the L3 group had an almost 2-fold increase and L4 had a 1.5-fold increase compared to L1 at the last sampled interval (Figure 7 for TP2 and TP3).

**Figure 8:**
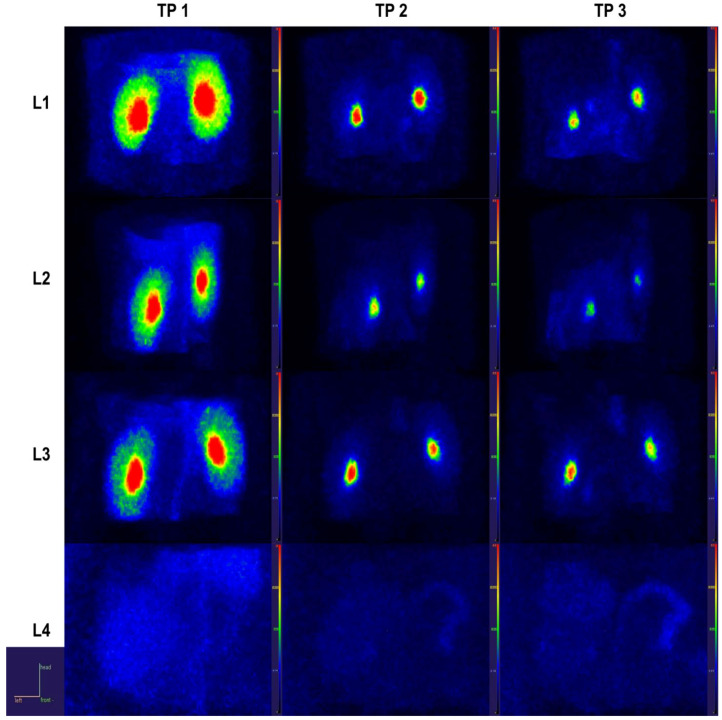
Renal close-ups in parametric maximal intensity projection images with voxel-wise normalization to blood SUV, depicting different behaviors of ^18^F-FDG according to time interval and group.

Renal cortical metabolic activity manifested a peculiar behavior, with a first-phase decrease in the distribution of radiotracer in L4 individuals compared to all other groups, followed by a marked elevation in the intermediary phase for L3 and L4 (Figure 8 for TP1 and TP2). By the time of the uptake-dominated phase, these alterations were reversed to the level of the L1 and L2 groups (Figure 8 for TP3). As a proxy for the renal function, the renal output was viewed as the pelvis normalized to cortical tracer concentration (Figure 6d) and displayed a relatively stable pattern across time intervals and groups, with the notable exception of L4 rats, in whom the output marker was severely blunted.

#### 2.4.2. Dynamic Data, Early Patlak on 1 s Reconstruction

Plotting the time–activity curves for each region, distinct periods of linearity could be observed, with each tissue’s corresponding plot with its own peculiarities. The cortex and medulla both had, in the first non-zero activity frames, a region of sharp linear increase that reached the peak, followed by a region that could be approximated as an exponential decay. The pelvis had in its first 60 s an exponential increase, a peak, and then an exponentially decreasing curve (probably a spillover artifact), followed by a linear increase that was of higher amplitude and with a higher peak. After this higher peak had been reached, the curve became highly non-linear, with particular shapes for each individual and with an overall decreasing tendency. The liver curve started with a long period of linearity, reaching the peak usually 1 min after the tracer administration.

**Figure 9:**
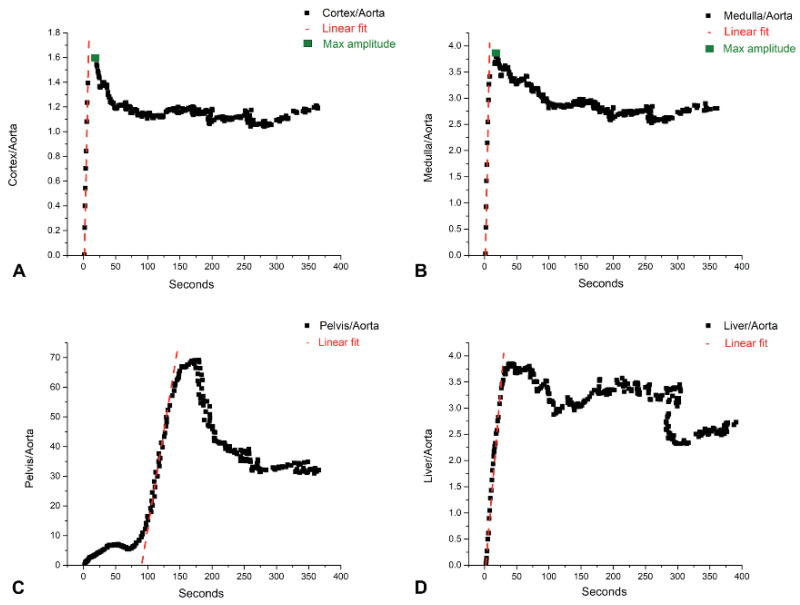
Plotted Patlak graphical analysis data, with examples represented for kidney cortex (**A**), medulla (**B**), pelvis (**C**), and liver (**D**), with superimposed linear fit for the analyzed duration and the maximum amplitude points.

**Figure 10:**
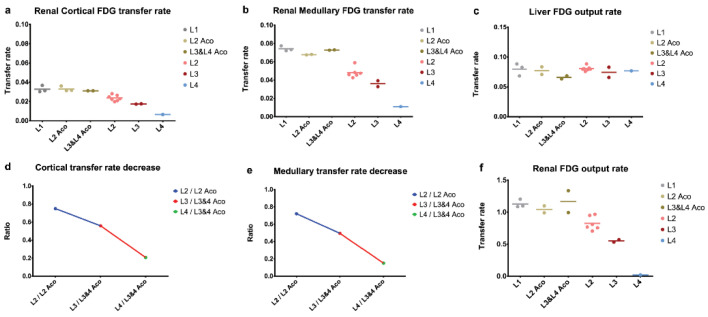
Patlak graphical analysis results for the cortex, medulla, pelvis (renal FDG output rate), and liver, with every individual depicted as a dot, and the line representing the average group value (**a**–**c**,**f**), together with the evolution of the cortex and medulla expressed as ratios, between L2, L3, and L4 and their respective age control groups (**d**,**e**).

The slope of the linear regression was used to devise a marker for each aorta-dependent tissue (Figure 9). Since for the cortex (Figure 9A) and medulla (Figure 9B), the slope of the Patlak plot was linear in the first few seconds of tissue activity, this marker would translate in physiological terms as a vascularization analog. For the pelvis (Figure 9C), on average, the analyzed region was about 60 s after the tracer administration, which would correspond to the beginning of urinary excretion, and would be a marker of the renal urinary FDG extraction rate. The liver marker (Figure 9D) was used as the control within each group and also for group comparisons. These distinct periods of linearity were found to be present in each of the subjects’ analyzed data and were used to extract the Ki as the slope of the linear regression. The obtained results are presented in Figure 10. This allowed for a strong differentiation between the groups, and a sizable difference when comparing L1, L2, L3, and L4 between them and with their corresponding age control individuals, respectively.

L1 and the healthy age control groups presented almost equal values when regarding the liver marker (Figure 10c), which we used as an internal control for the employed method. In contrast, L2, L3, and L4 presented a general value decrease with regard to the cortical (Figure 10a), medullary (Figure 10b), and pelvic markers (Figure 10f), as also seen in the ratio of these tissues (Figure 10d,e), from their respective age controls.

#### 2.4.3. Dynamic Data and Compartmental Model

**Figure 11:**
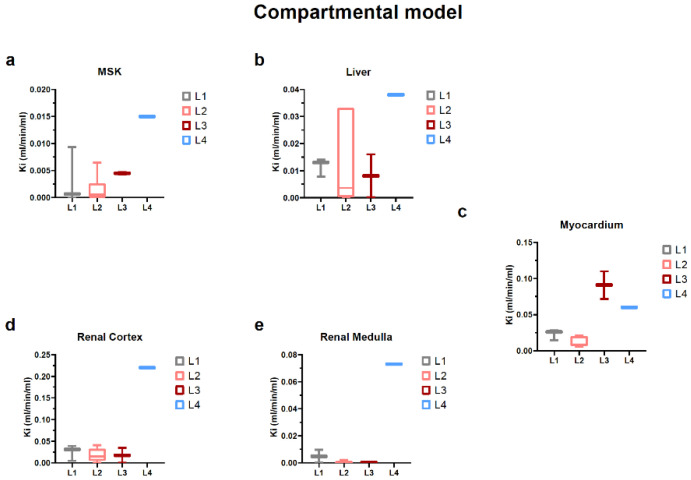
K_i_ values derived from compartmental model for each target organ/region and also for internal control organs, namely skeletal muscle (**a**), liver (**b**), myocardium (**c**), renal cortex (**d**) and renal medulla (**e**).

This task of deriving specific 18F-FDG net uptake rates (Ki) from time–activity curves was approached by means of compartmental modeling for the early non-linear phase of the tracer distribution. Skeletal muscle Ki in the L3 group was in the upper range of the control and L2 groups, whereas for L4, the ^18^F-FDG uptake rate had a 3-fold increase (Figure 11a). In the case of the liver, L3 rats did not differ from the control, while the wide distribution of L2 values was surpassed only by the L4 group (Figure 11b). The net influx rate for the heart muscle mirrored the pattern of distribution seen in static data across the groups (Figure 11c). In contrast, the Ki for renal cortex in L3 did not reflect the intermediary phase increase noticed in the static data analysis, whereas this seems not to be the case for the L4 group (Figure 11d). In an attempt to delineate the possible distinct tracer handling by the renal parenchyma, the cortical was separated from the medullary region, and while medullary Ki values were generally lower, the between-group patterns did not differ (Figure 11d,e).

## 3. Discussion

In conditions of normoxemia and normal substrate availability, the myocardial muscle relies heavily on fatty acid oxidative metabolism with moderate contributions from aerobic glycolysis and intermediary products such as lactate and ketone bodies [32,33]. In response to chronic contractile failure, this metabolic redundancy pays off since an increase in oxidative glycolysis provides a global improvement in energetic efficiency by about 30%. Heart failure studies, in both rodent [34] and human subjects [35], are consistently pointing to a progressive transition from fatty acid β-oxidation to glucose and ketone bodies as the preferred substrates for energetic needs, but also for defending against free radical species generated.

Doxorubicin is a cationic drug that binds with high affinity to cardiolipin (a phospholipid), forming an almost irreversible complex in the inner mitochondrial membrane [36]. The evidence suggests that it disrupts the cardiolipin–protein interface, causing the formation of several reactive oxygen species [36]. In turn, these free radicals damage the mitochondrial function and result in altered energy metabolism [37]. Moreover, reactive oxygen species alter normal calcium homeostasis by disrupting the function of the sarcoplasmic reticulum [38]. At the same time, doxorubicin inhibits the opening of the sodium-calcium channels in the plasma membrane, leading to mitochondrial dysfunction and apoptosis [38]. The administration of doses above 1 mg/kg results in a lower survival rate and classic dilatative cardiomyopathy signs [39]. Nephrotoxicity and hepatotoxicity are also significant side effects in addition to the well-known cardiotoxicity, and this fact imposes certain limitations on the clinical use of doxorubicin [40,41,42].

As a measure of general toxicity, the growth trajectories of L2 and L3 rats and doxorubicin-treated groups, when compared to the controls, are almost in superposition, but their growth rate is shifted toward the lower end of the distribution seen in healthy rats, suggesting an effect of developmental stunting. It is well known that a cumulative dosage of up to 12 mg/kg of doxorubicin demonstrates a spectrum of body weight alterations after exposure, depending on the dose, rate, and formulation of the agent. The dosing schedule applied in our study, of 1 mg/kg twice per week for 6 weeks [20], seems to have a minimal impact on general growth, in agreement with the findings of Storm et al. [43].

In cultured cardiomyocytes [44] and in vivo [45], doxorubicin-induced oxidative stress is reflected by the activation of apoptotic transcriptional networks and the downregulation of mitochondrial biogenesis markers, PGC-1α and PGC-1β. In a rat model similar to this study [8], a link between Na-dependent glucose transporter (SGLT) inhibitors, empagliflozin, and the reduction in cell apoptosis/mitochondrial dysfunction suggest the adverse role of anaerobic glycolytic pathways in the CRS syndrome. As markers of myocardial ventricular stress, natriuretic peptides (e.g., NT-proBNP) provide an early, preclinical index of ventricular dysfunction and a sensitive tool in clinical practice for discriminating heart failure severity [46]. Under the conditions of our study, we did not observe any marked departures of NT-proBNP serum levels in L2 and L3 groups or doxorubicin-only treated rats in comparison to control rats. This finding is in contrast to other rat studies using similar doxorubicin regimens [20], and since other tests (growth rate, metabolic cage monitoring, pathology examination, and ^18^F-FDG PET imaging) are indicative of myocardial alterations, we envision two possible explanations. One of these could be related to assay reliability, but the standard curves were in agreement with the kit manufacturer’s specifications. A second potential explanation could claim a lack of sufficient volume overload on the ventricular walls [47,48].

Rodent model studies [49], but also human biopsies for chronic kidney disease [50], provide evidence for dysfunctional mitochondrial oxidative processes and the up-regulation of glycolytic pathways, mainly in response to alterations in oxygen diffusion due to tubulointerstitial fibrosis. Despite the extensive proof of doxorubicin-induced nephrotic syndrome pathology in rodent studies and the progression of chronic kidney disease as a function of time, conventional serum markers (creatinine, urea) are highly dependent on doxorubicin dosage, the schedule of administration, timing of blood sampling, and nutritional composition of food [51]. Within the dosing regimen used in the present study, with a cumulative dose of 12 mg/kg over 6 weeks, serum creatinine and urea, as markers of glomerular filter injury, did not present any deviations from the control group in doxorubicin-only treated rats, only in those pre-treated by 5/6 nephrectomy. These observations are in agreement with other preclinical studies using similar doxorubicin regimens [52].

Conversely, in the case of the renal tubular injury marker, neutrophil gelatinase-associated lipocalin increased urinary levels measured in L3 versus L2 individuals, suggesting a time-dependent process of tubular toxicity based on the slow excretion pharmacokinetics and tissue biodistribution of doxorubicin [53]. In a similar vein, the lack of difference between L2 and the control rats implies the existence of a necessary renal cumulative dose threshold for progression to actual tubular epithelial injury [54].

Following renal mass reduction by subtotal nephrectomy, tissue pathology examinations exposed the relationship between hemodynamically modified nephrons and chronic renal injury [55]. After subtotal nephrectomy, the synthesis and storage of intraglomerular renin were increased in glomeruli and areas adjacent to the infarct site [56]. The proteomic analysis of the particular 5/6 nephrectomy model we employed demonstrates the downregulation of mitochondrial oxidative enzymatic networks with the increased turnover of mitochondria. Moreover, plasma and urinary metabolomic studies [57] are consistent in proving an increased metabolic disorder in this model of chronic kidney disease.

The simultaneous induction of a cardiorenal syndrome rat model leads to the significant additive damage of doxorubicin to the renal function under 5/6 nephrectomy conditions, shortening the time course to overt proteinuria and severe tubulointerstitial fibrosis [58]. In our study and in contrast to the L3 group, the additive effect of doxorubicin in L4, 5/6 nephrectomy pretreated rats was observed, mainly through the concurrent elevations of serum markers of glomerular dysfunction (creatinine, urea), in parallel to NGAL. While myocardial failure marker levels, NT-proBNP, did not suggest any consequences of chronic uremia on cardiac function compared to L2 and L3, it is experimentally shown that 5/6 nephrectomy leads to systemic arterial hypertension and left ventricle structural and functional remodeling [12].

Caspase-3 is involved in the pathogenesis of doxorubicin-induced cardiomyopathy [59,60] and nephropathy [61]. As a more proximal step in the cell response to doxorubicin toxicity, p53 overexpression plays a major role in developing dilatative cardiomyopathy and chronic kidney disease [11] mainly through its’ genomic integrity monitoring activity [62].

At the distal end of the chain of events induced by chemotoxicity and tissular lesions, α-SMA, as a marker of fibroblast activation, reflects the reactive consequences of tissue remodeling following extensive apoptosis under doxorubicin exposure in the myocardium [63] and kidney [64]. As a model of chronic renal failure, 5/6 nephrectomized rats demonstrate a potent induction of interstitial myofibroblast activity in the remnant kidney mass [65], but as uremic toxins accumulate, this is also observed at the level of the cardiac tissue [66].

Across the examined groups in our study, in L2 and L3, but also L4, a pattern of the progressive expression of the above-mentioned immunohistochemistry markers of tissue injury was observed. Regarding myocardial histopathological alterations, there was a close overlap between areas of myofibrils degeneration, the density of intracytoplasmic vacuolization, and the extent of positive apoptosis and fibrosis markers. At the kidney level, the distribution of glomerular lesions manifested as sclerosis, Bowmann capsule thickening, and fusion followed the increasing pattern of caspase-3 and p53 expression, while areas with tubular atrophy and proteinaceous casts displayed additional interstitial α-SMA positivity.

As stated previously, myocardial metabolism is a highly substrate-dependent process, with a high uptake of glucose analogs during postprandial states [67]. During myocardial cell chemotoxic insult, the emerging mitochondrial dysfunction translates into a metabolic switch from fatty acid oxidation to anaerobic glycolysis [68]. In our study, due to food deprivation prior to FDG-PET examination, we observed a global absence of the low uptake of the radiotracer in the cardiac muscle of control and early doxorubicin groups. The uptake in skeletal muscle and liver, as an internal reference for the general metabolic milieu, demonstrated a reliable background in assessing myocardial alterations in glucose metabolism.

In delayed doxorubicin-treated and subtotal nephrectomy preconditioned rats, myocardial toxicity seemed to pass the necessary threshold, with sizable elevations of the tracer uptake compared to the first two groups. Moreover, the L3 group displayed a stable metabolic background milieu, whereas, for L4 rats, there was an apparent upregulation in glycolytic activity in the skeletal muscle and liver, suggesting the systemic compounding effects produced by uremic toxins and doxorubicin. While this interpretation stems from dynamic PET data modeling results, when looking for corroboration with static PET data during late time points (i.e., past 20 min after radiotracer injection), we did not find an agreement on increased uptake ratio for these organs in the L4 group. This difference suggests alterations mainly in the vascular function and/or extracellular volume but not actual intracellular glucose phosphorylation.

In healthy states, 2-deoxy-glucose handling on the tubular luminal pole after glomerular filtration follows the same pathways as endogenous glucose, but with a different pharmacokinetic rate profile (low affinity for SGLT) and prior to filtration, at the level of the vascular pole, there was an insulin-dependent uptake due to the distribution of glucose transporters for transepithelial reabsorption processes [69]. On top of that, the renal interstitial space is also host to a significant fraction of immune and stromal cells [70], and since 2-deoxy-glucose is a good marker of inflammatory aggregates [71] and fibroblast activation [50], this may act as a possible confounder in interpreting the global uptake rates (i.e., Ki).

Despite histopathology showing an increased frequency of inflammatory infiltrates from L1 to L4 groups in the renal specimens, only L4 rats manifested a marked elevation in the net uptake rates of ^18^F-FDG. While the source of this increase in tracer uptake was obscured by the non-specific nature of ^18^F-FDG distribution regarding cell types, the confluence of processes in advanced renal failure leads to the global retention of the radiotracer in the parenchyma. Taking into account the high and relatively quick filtration of FDG at the kidney level and its distribution into other body organs, it has been a difficult endeavor to determine a good PET marker for renal injury or find a renal function surrogate.

The discussion of the early Patlak analysis (1s frame reconstruction) was conducted by referencing the renal pathology exam as the established procedure in determining histological alterations, and the biochemical exam, through urea and creatinine, as the conventional procedure in determining renal function alterations. Histologically, clear tissue and structural damage were proven, with obvious aggravation from L2 to L4. From this perspective, we can conclude that the subjects investigated by PET/MRI had a progressive structural kidney alteration from L2, L3, to L4, with L1 exhibiting normal parameters.

Biochemically, urea and creatinine were over the pathological threshold only in L4 (Figure 2); thus, it can be concluded that observable kidney disease would occur only in the most affected subject lot. Urea and creatinine increase at a pathological level only when at least 50% of the kidney parenchymal structure is altered [72]. Thus, we could assume that if the dynamic PET data analysis shows differentiation between L1, L2, L3, and L4, it would be more sensitive than creatinine to present or even future renal function alteration. In this regard, we believe that we had, through PET/MR study, a valuable and not a commonly found cohort of individuals that exhibited progressive histological renal alterations that could be compared with healthy individuals.

A discriminatory marker of renal impairment should be one that matches or surpasses the capability of the accepted blood creatinine and urea. Therefore, we could state that any discrimination obtained outside that of L1, L2Aco, L3&4Aco, as is the case in L2, L3 vs. L4, could be seen as a predictor of present or future renal function alterations. The concept underlying the use of the aorta activity as the input function and the renal pelvis as the irreversible compartment is helpful in bypassing the complex and simultaneous processes that involve the passing of FDG from the vascular into glomerular space, and from there, through renal processes that lead to FDG urinary excretion.

For the parenchymal region of the kidney, the time interval in which the slope of the linear regression was extracted (essentially the influx rate constant) started from the first non-zero activity frame and ended about 9–10 s thereafter (Figure 9). The short duration at the beginning of the administration would be suggestive of the first phase of the tracer dynamic; the vascular distribution and fluctuations in its values would indicate a vascular transit alteration. Comparing the marker derived from this tissue by each group, we could observe that L1 and the individuals from the age control groups maintained similar values (Figure 10), which indicates that there were no evident changes if compared to the healthy rats at different ages. We could also observe a decrease in the cortex and medullary vascular markers when comparing the healthy subjects to L2, L3, or L4 (Figure 11), presented also as a ratio of the L2, L3, L4 cortical or medullary markers to their respective age controls. These values decrease in a progressive manner, in line with the progressive histopathological alterations.

The renal total blood flow is remarkably stable through large variations of the arterial blood pressure from 100 to 200 mmHg. As a consequence, the impact on the vascularization from the administration of the intravenous FDG bolus should be negligible. We believe that the lower amplitude and lower slope of the first 9 s of the FDG passage of the cortex curve, compared to the medulla, could be attributed to the higher caliber vessels and higher capillary network and density that are found at this level [73]. Although the blood flow is 80–90% to the cortex and only 10% to the medulla of the total kidney blood volume, we measured the blood that passed through these two regions and not their respective blood flow distribution, which differ substantially.

An interesting observation could be made about the peak amplitudes of the cortical and medullary curves in that the cortical peak amplitude is approx. two times lower (0.48) for the healthy rats than the medullary one, and in treated rats, the cortical to medullary amplitude ratio increases slightly in value throughout, L2 (0.48), L3 (0.52), and L4 (0.53) (mostly on the cortex uptake decreasing), suggesting a more than double tracer uptake in the medullary compared to the cortex in this vascular window. This vascular space alteration and transfer rate alterations could be an indicator of the present or future development of renal dysfunction [74].

For the pelvic marker, the time interval on which the slope was derived started from approx. 60 s after the first non-zero frame until about 120 s (Figure 9c). The subsequent marker could be viewed as encompassing the ramp-up of urinary FDG excretion. Thus, the value of it could be as a surrogate of kidney efficiency to transport vascular FDG to the urine and encompassing all processes that led to urinary excretion, ultimately being interpreted as an extraction marker. This marker would be the closest that could describe the kidney function by one value that we could derive by this method. Comparing L1 and the age control groups, we could see that the values do not differ notably, making this marker somewhat stable and non-evolving with age. When the comparison is made with L2, L3, and L4, a noticeable value decrease can be observed. This will lead us to conclude that the extraction fraction decreases as the histopathological alterations increase, and the age in healthy individuals has no influence on it (Figure 10f).

Taking into account that the rats were studied during their growth period, and kidney dimensions should have followed their growth curve, the inverse variation with age of the renal volumes, or its stagnation, is a counterintuitive finding (Figure 5). Potter et al. [75] conducted morphological studies showing that, until 16 weeks old, the kidney volume increase was primarily based on cellular multiplication and less on the increase in its volume, and after 16 weeks, it was based primarily on cell volume increase and less on cell multiplication. Therefore, this phenomenon probably can be explained by the renal toxicity of the repeated doxorubicin administrations and its toxic DNA and mitochondrial effects.

## 4. Materials and Methods

### 4.1. Study Population

The experimental protocol was approved by the Ethical Committee of Grigore T. Popa University of Medicine and Pharmacy of Iasi in accordance with Directive 2010/63/EU, taking into consideration the 3R principles.

**Figure 12:**
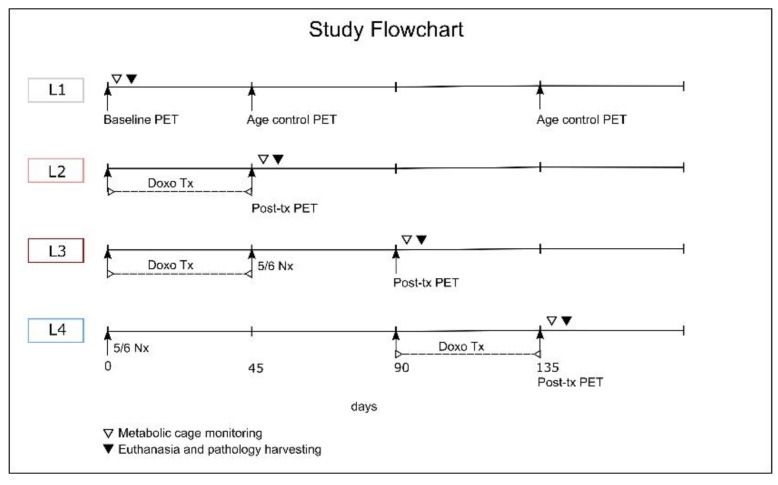
Study flow diagram for the interventions and examinations for each group under study in time.

A total of 34 Wistar albino male rats (2 months old at arrival) were divided into 4 groups (Figure 12): 8 animals in the control (L1), 8 in doxorubicin-induced heart failure (L2), 8 in cardiorenal syndrome (L3) and 10 in renocardiac syndrome (L4). On top of that, 4 rats were kept alive as the age control of the 18F-FDG PET imaging data, annotated as L2Aco for L2 and L3&L4Aco for L3 and L4. They were housed in individually ventilated cages containing shaving bedding material, with a diet consisting of 10 g standard rodent chow per day and water ad libitum, with a 2-week period of acclimatization. The facility was climate-controlled: 20 ± 4 °C, 50 ± 5% relative humidity, and 12 h light/dark cycles. The interquartile range for body weight at study entry for each group was 293–324 g for L1, 290–297 g for L2, 278–321 g for L3, and 282–319 g for L4.

### 4.2. Doxorubicin and Renal Mass Reduction Protocols

Cardiac dysfunction was induced by the intraperitoneal injection of 1 mg/kg of doxorubicin (Doxorubicin Hydrochloride, 2 mg/mL, Accord^®^, Ireland, Cork), twice a week for 6 weeks, following a protocol adapted from Shen et al. [20].

Renal failure was induced by the 5/6 nephrectomy protocol, following the guidelines of Zhang & Kompa [21]. After proper animal acclimatization and prior surgery temperature-controlled bed setup, anesthesia was induced with 5% isoflurane and maintained at 2% for the rest of the procedure. The decontaminated and shaved abdominal region was incised on the midline and aided by sterile retractors and cotton buds; a deep dissection for the kidney pedicle was performed. Left posterior and inferior kidney arteries were ligated (confirmed by ischemic discoloration in 2/3 of the parenchyma), and excision of the right kidney was made en bloc. The closure of the abdominal wall was attained after the control of hemostasis and cavity wash with 3 mL of serum. All surgical procedures were performed in a sterile manner.

### 4.3. Clinical, Metabolic Cage Monitoring and Biochemistry

During the observation period, each cage was inspected on a twice-per-week basis, checking for signs of abnormality in behavior or appearance without interfering with the habitat. Afterward, the rats were weighed, extremities were inspected for abnormal pigmentation or dystrophies, and skin turgor was assessed as a marker of extracellular fluid accumulation.

Due to particular study design constraints (detailed in the Study limitations section), longitudinal body weights for the control group were derived from the literature data [22] and synthesized by a 5-point sigmoidal function. Growth rates were estimated by linear regression of the natural log-transformed weights on the number of postnatal days.

At the end of the PET experiments (as pointed out in Figure 12), each rat was placed in a metabolic cage for a monitoring period of 24 h. Food intake, urine, and feces output were measured, and results were normalized to body weight. This was followed by euthanasia with blood and target organ sampling.

Creatinine and urea were determined at the end of the study from the serum samples by the automated biochemical analyzer. Creatinine was determined by the Jaffé kinetic method (colorimetric), while the urea was assessed spectrophotometrically. The urinary marker of tubular injury (NGAL, Rat Lipocalin-2/NGAL, Fine Test, cat. no. ER0003, Wuhan, China) and NT-ProBNP (N-Terminal Pro-Brain Natriuretic Peptide, Fine Test, cat. no. ER0309, Wuhan, China) was determined by the ELISA method, according to the manufacturer’s kit protocol.

### 4.4. PET/MRI Data Acquisition

Each rat, fasted overnight, was brought from the biobase on the morning of the experiment and left to acclimatize to the scanner at room temperature for an average of 1 h. Anesthesia was induced by an isoflurane delivery system connected to a transparent chamber at a concentration of 5% isoflurane in a mixture of air and oxygen with a flow of 3 L/min. After induction, the animal was transferred to the scanner bed of the nanoScan PET/MRI (Mediso^®^, Budapest, Hungary) with a full width at half maximum of 0.9 mm and detection sensitivity [23] of 42.8 cps/kBq, in a prone position and maintenance anesthesia was adjusted to 3% isoflurane with a flow of 1 L/min.

A fast MRI scout for system calibration and subject localization was performed. It was followed by an axial T1-weighted 3D spoiled gradient recalled echo sequence (GRE) for anatomical referencing (slice thickness = 2 mm, TR = 10.6 ms, TE = 3.2 ms, NEX = 4, flip angle = 15°). Before starting PET data acquisition, the animal was brought out of the MRI coil, and the tail vein was cannulated with the aid of a warm water bowl. After bringing the bed inside the PET ring and initiating data acquisition, ^18^F-FDG (manufactured at a regional radiopharmacy) was administered through a 26G cannula (average dose of 9.56 ± 0.7 MBq/100 g). Pre-scan glycemia levels were measured with a clinical-grade glucometer before ^18^F-FDG administrations.

PET data acquisition consisted of a 40 min dynamic scan with a single FOV over the thorax and abdominal region without cardiac gating. Data acquisition was in list mode, 3D, with a 1 to 5 in-plane coincidence detection activated and a normal photon count rate.

Data reconstruction employed the proprietary Tera-Tomo engine (Mediso^©^) with a 3D maximum a posteriori algorithm (8 iterations and 3 subsets) using an energy window of 400–600 keV, a 0.6 mm isotropic voxel size, a small regularization term (alpha = 0.0001 and beta = e^−3^, and a variance-reduced delayed window for random correction. Photon attenuation and scatter correction of the data were considered unnecessary. List-mode data were partitioned into a dynamic study for the first 5 min with a high-frequency sampling of 1 s, the next 15 min with 10 s per frame, and the last 20 min with 30 s per frame. Additionally, 3 static reconstructions with a 5 min interval each were performed for the standard uptake value (SUV) analysis for 0–5 (TP1), 15–20 (TP2), and 35–40 (TP3) minute periods.

### 4.5. PET Data Extraction and Analysis

PET data analysis was conducted by two independent evaluators in two separate data extraction software environments: Carimas (Turku PET Centre^©^, Turku, Finland) and Vivoquant™ (InviCRO^©^, London, UK). As the first step of segmentation, the PET and MR images were rescaled and merged. Averaged dynamic PET and MR images were reviewed for proper co-registration by manual adjustments on a region-by-region basis according to the available data [24,25,26,27,28]. Volumes of interest (VOI) were drawn according to Figure S1. More details can be found in Supplementary Materials.

### 4.6. Histopathology and Immunohistochemistry Analysis

The kidneys and heart were fixed in a 10% formalin solution and refrigerated until pathology processing. The organs were properly trimmed and embedded in paraffin. Tissue sections with a 5 μm slice thickness were stained with hematoxyllin-eosin. Tissue slides were labeled for immunohistochemistry (IHC) with caspase-3 (apoptosis marker), p53 (genotoxicity marker), and α-sma (hypertrophy/fibroblast activation marker). The procedure was initiated with deparaffinization in xylene and then rehydration through a descending gradient of ethanol. After that, the slides were incubated with hydrogen peroxide to remove the endogenous peroxidase activity. Non-specific binding was blocked by incubation with a blocking buffer for 15 min. The sections were then incubated overnight with anti-caspase-3 (1:200 diluted with standard phosphate-buffered solution (PBS), p53 (1:100 diluted with PBS), α-sma (1:500 diluted with PBS). The antigen–antibody complexes were visualized as a brown color using an avidin-biotin-peroxidase complex solution and using a Vectastain^®^ ABC Kit (Vector inc., Torrance, CA, USA). Sections were developed with 3,3′-diaminobenzidine (DAB) and finally counter-stained with hematoxylin.

All tissue slides were examined without prior knowledge of the control and intervention groups. Myocardial tissue slides were examined qualitatively for intracytoplasmic vacuolization, the depletion of myofibrils, areas of necrosis, and areas of inflammatory infiltration, while the renal slides were assessed for areas of glomerular sclerosis, tubular atrophy, proteinaceous casts, and inflammatory changes.

### 4.7. Statistical Analysis

The data analysis results are described by box plots with a whole min-max range for each experimental group. Additionally, for body weight monitoring, a connected line plot was used with a median and 95% confidence interval at each data point in time, while for survival analysis, a Kaplan–Meier plot was applied. Due to the small sample size in target groups (L3, L4) and a large number of target variables, either exploratory statistical techniques or hypothesis testing for effect size significance were deemed inappropriate.

## 5. Conclusions

In our particular model of the cardiorenal syndrome, chronic treatment with low-dose doxorubicin followed by acute 5/6 nephrectomy incurred the highest mortality among the study groups, while the model for renocardiac syndrome resulted in moderate-to-high mortality. Doxorubicin cardiotoxicity was clearly identified by ^18^F-FDG PET imaging, not at the end of regimen administration, but after a period of another 6 weeks. Pre-treatment by 5/6 nephrectomy at 3 months before starting the doxorubicin regimen nulled any compensatory reserves with ensuing cardiac metabolic abnormality. Doxorubicin administered during the growth period impaired normal kidney development. It appears that ^18^F-FDG PET could be sensible to vascular alterations seen in the progressively altered kidney and could also give possible insight into its function.

Given the fact that standard clinical markers were insensitive to early renal injury, we believe that the decreasing values of the ^18^F-FDG PET-derived renal marker across the groups, compared with their age-matched controls, and along with the uniform distribution seen in healthy developing rats, could have a potential diagnostic and prognostic yield in cardiorenal syndrome.

## 6. Study Limitations

Due to the nature of the study, i.e., a preclinical small animal experimental study and 3R bioethical constraints, and the lack of previous experimental work on this particular model of cardiac and renal failure during study design and preparation, we encountered several limitations in the data collection, analysis and interpretation, briefly described below:

- The high mortality rate among L3 and L4 individuals produced a small sample size and the inability to repeat the experiment if they exhibited tail radiotracer trapping; also, due to financial and bioethical restrictions, we did not have the opportunity to gain more insight into the timeline of model pathophysiology through a repeated sampling of the salient biochemical, histological or imaging markers, at multiple points in time, for the same group of rats (Figure S2).
- Due to the small sample size in target groups (L3, L4) and a large number of target variables, neither the exploratory statistical technique nor hypothesis testing for effect size significance was performed.
- From the perspective of PET/MRI imaging, a serious limitation was the lack of any modality to gate the PET and MR signal to cardiac or respiratory motion due to technical issues, which posed additional difficulties in image data extraction and interpretation. In addition, the limited bore size of the MRI RF antenna made it impossible to accommodate the oversized ascitic L4 rats for T1 anatomical sequencing.
- Another restriction related to the PET/MRI system was an inaccuracy of the scanner bed positioning between the PET and MRI modalities due to the peculiar geometry of the bed, which generated significant challenges in fusing PET and MRI studies.
- During data acquisition and analysis, about ¼ of the subject rats had almost complete tracer trapping in the tail vein upon injection, which led to the exclusion of these individuals from the PET analysis, and subsequently reduced our subject number.
- Due to the aforementioned lack of respiratory gating, limited bore size, and the particular remodeling of the renal parenchyma for L4 rats after surgery meant that the delineation of the renal pelvic region was severely impaired or even impeded in some.
- Regarding subject eligibility for analysis, some individuals exhibited bilateral renal pelvic abnormalities, leading to their exclusion from the PET analysis, the curves derived from this region being incomparable with the ones derived from the other subjects.

## Data Availability

The datasets used and/or analysed during the current study are available from the corresponding author on reasonable request, namely Dr. Gianina Dodi at gianina.dodi@umfiasi.ro.

## Supplementary Materials

The following supporting information can be downloaded at: https://www.mdpi.com/article/10.3390/ijms232315409/s1.

## References

[1] Ronco C., McCullough P., Anker S.D., Anand I., Aspromonte N., Bagshaw S.M., Bellomo R., Berl T., Bobek I., Cruz D.N. (2009). Cardio-renal syndromes: Report from the consensus conference of the Acute Dialysis Quality Initiative. Eur. Heart J..

[2] Kumar U., Wettersten N., Garimella P.S. (2019). Cardiorenal Syndrome. Cardiol. Clin..

[3] Grossman R.C. (2010). Experimental Models of Renal Disease and the Cardiovascular System. Open Cardiovasc. Med. J..

[4] Chang D., Wang Y.C., Zhang S.J., Bai Y.Y., Liu D.F., Zang F.C., Wang G., Wang B., Ju S. (2015). Visualizing myocardial inflammation in a rat model of type 4 cardiorenal syndrome by dual-modality molecular imaging. Biomaterials.

[5] Chuppa S., Liang M., Liu P., Liu Y., Casati M.C., Cowley A.W., Patullo L., Kriegel A.J. (2017). MicroRNA-21 regulates peroxisome proliferator–activated receptor alpha, a molecular mechanism of cardiac pathology in Cardiorenal Syndrome Type 4. Kidney Int..

[6] Melenovsky V., Cervenka L., Viklicky O., Franekova J., Havlenova T., Behounek M., Chmel M., Petrak J. (2018). Kidney Response to Heart Failure: Proteomic Analysis of Cardiorenal Syndrome. Kidney Blood Press. Res..

[7] Zhao Y., Wang C., Hong X., Miao J., Liao Y., Hou F.F., Zhou L., Liu Y. (2019). Wnt/β-catenin signaling mediates both heart and kidney injury in type 2 cardiorenal syndrome. Kidney Int..

[8] Yang C.-C., Chen Y.-T., Wallace C.G., Chen K.-H., Cheng B.-C., Sung P.-H., Li Y.-C., Ko S.-F., Chang H.-W., Yip H.-K. (2018). Early administration of empagliflozin preserved heart function in cardiorenal syndrome in rat. Biomed. Pharmacother..

[9] Giam B., Kaye D.M., Rajapakse N.W. (2016). Role of Renal Oxidative Stress in the Pathogenesis of the Cardiorenal Syndrome. Heart Lung Circ..

[10] Chua S., Lee F.-Y., Chiang H.-J., Chen K.-H., Lu H.-I., Chen Y.-T., Yang C.-C., Lin K.-C., Chen Y.-L., Kao G.-S. (2016). The cardioprotective effect of melatonin and exendin-4 treatment in a rat model of cardiorenal syndrome. J. Pineal Res..

[11] Lahoti T.S., Patel D., Thekkemadom V., Beckett R., Ray S.D. (2012). Doxorubicin-Induced In Vivo Nephrotoxicity Involves Oxidative Stress- Mediated Multiple Pro- and Anti-Apoptotic Signaling Pathways. Curr. Neurovasc. Res..

[12] Svíglerová J., Kuncová J., Nalos L., Tonar Z., Rajdl D., Stengl M. (2010). Cardiovascular parameters in rat model of chronic renal failure induced by subtotal nephrectomy. Physiol. Res..

[13] Dilsizian V., Arrighi J.A., Diodati J.G., Quyyumi A.A., Alavi K., Bacharach S.L., Marin-Neto J.A., Katsiyiannis P.T., Bonow R.O. (1994). Myocardial viability in patients with chronic coronary artery disease. Comparison of 99mTc-sestamibi with thallium reinjection and [18F]fluorodeoxyglucose. Circulation.

[14] Youssef G., Leung E., Mylonas I., Nery P., Williams K., Wisenberg G., Gulenchyn K.Y., Dekemp R.A., Dasilva J., Birnie D. (2012). The Use of 18F-FDG PET in the Diagnosis of Cardiac Sarcoidosis: A Systematic Review and Metaanalysis Including the Ontario Experience. J. Nucl. Med..

[15] Saby L., Laas O., Habib G., Cammilleri S., Mancini J., Tessonnier L., Casalta J.-P., Gouriet F., Riberi A., Avierinos J.-F. (2013). Positron Emission Tomography/Computed Tomography for Diagnosis of Prosthetic Valve Endocarditis. J. Am. Coll. Cardiol..

[16] Kandolin R.M., Wiefels C.C., Mesquita C.T., Chong A.-Y., Boland P., Glineur D., Sun L., Beanlands R.S., Mielniczuk L.M. (2019). The Current Role of Viability Imaging to Guide Revascularization and Therapy Decisions in Patients with Heart Failure and Reduced Left Ventricular Function. Can. J. Cardiol..

[17] Terrovitis J., Lautamäki R., Bonios M., Fox J., Engles J.M., Yu J., Leppo M.K., Pomper M.G., Wahl R.L., Seidel J. (2009). Noninvasive Quantification and Optimization of Acute Cell Retention by In Vivo Positron Emission Tomography After Intramyocardial Cardiac-Derived Stem Cell Delivery. J. Am. Coll. Cardiol..

[18] Geist B.K., Baltzer P., Fueger B., Hamboeck M., Nakuz T., Papp L., Rasul S., Sundar L.K.S., Hacker M., Staudenherz A. (2018). Assessing the kidney function parameters glomerular filtration rate and effective renal plasma flow with dynamic FDG-PET/MRI in healthy subjects. EJNMMI Res..

[19] Garbarino S., Caviglia G., Sambuceti G., Benvenuto F., Piana M. (2014). A novel description of FDG excretion in the renal system: Application to metformin-treated models. Phys. Med. Biol..

[20] Shen L.-j., Lu S., Zhou Y.-h., Li L., Xing Q.-m., Xu Y.-l. (2016). Developing a rat model of dilated cardiomyopathy with improved survival. J. Zhejiang Univ.-Sci. B.

[21] Zhang Y., Kompa A.R. (2014). A practical guide to subtotal nephrectomy in the rat with subsequent methodology for assessing renal and cardiac function. Nephrology.

[22] Nistiar F., Racz O., Lukacinova A., Hubkova B., Novakova J., Lovasova E., Sedlakova E. (2012). Age dependency on some physiological and biochemical parameters of male Wistar rats in controlled environment. J. Environ. Sci. Health Part A.

[23] Nagy K., Tóth M., Major P., Patay G., Egri G., Häggkvist J., Varrone A., Farde L., Halldin C., Gulyás B. (2013). Performance Evaluation of the Small-Animal nanoScan PET/MRI System. J. Nucl. Med..

[24] Lanz B., Poitry-Yamate C., Gruetter R. (2014). Image-Derived Input Function from the Vena Cava for 18F-FDG PET Studies in Rats and Mice. J. Nucl. Med..

[25] Huang Q., Massey J.C., Mińczuk K., Li J., Kundu B.K. (2019). Non-invasive determination of blood input function to compute rate of myocardial glucose uptake from dynamic FDG PET images of rat heart in vivo: Comparative study between the inferior vena cava and the left ventricular blood pool with spill over and partial volume corrections. Phys. Med. Biol..

[26] Bertoldo A., Vicini P., Sambuceti G., Lammertsma A.A., Parodi O., Cobelli C. (1998). Evaluation of compartmental and spectral analysis models of [18/F]FDG kinetics for heart and brain studies with PET. IEEE Trans. Biomed. Eng..

[27] Kukreja S.L., Gunn R.N. (2004). Bootstrapped DEPICT for error estimation in PET functional imaging. NeuroImage.

[28] Doenst T. (1998). Complexities Underlying the Quantitative Determination of Myocardial Glucose Uptake with 2-Deoxyglucose. J. Mol. Cell Cardiol..

[29] Werner R.A., Hess A., Koenig T., Diekmann J., Derlin T., Melk A., Thackeray J.T., Bauersachs J., Bengel F.M. (2021). Molecular imaging of inflammation crosstalk along the cardio-renal axis following acute myocardial infarction. Theranostics.

[30] Sano Y., Ito S., Yoneda M., Nagasawa K., Matsuura N., Yamada Y., Uchinaka A., Bando Y.K., Murohara T., Nagata K. (2016). Effects of various types of anesthesia on hemodynamics cardiac function, and glucose and lipid metabolism in rats. Am. J. Physiol.-Heart Circ. Physiol..

[31] Tse F.L.S., Chang T., Finkelstein B., Ballard F., Jaffe J.M. (1984). Influence of Mode of Intravenous Administration and Blood Sample Collection on Rat Pharmacokinetic Data. J. Pharm. Sci..

[32] Brown D.A., Perry J.B., Allen D.A.B.J.B.P.M.E., Sabbah H.N., Stauffer B.L., Shaikh S.R., Cleland J.G.F., Colucci W.S., Butler J., Voors A.A. (2016). Mitochondrial function as a therapeutic target in heart failure. Nat. Rev. Cardiol..

[33] Bastiaansen J.A.M., Merritt M.E., Comment A. (2016). Measuring changes in substrate utilization in the myocardium in response to fasting using hyperpolarized [1-13C] butyrate and [1-13C] pyruvate. Sci. Rep..

[34] Aubert G., Martin O.J., Horton J.L., Lai L., Vega R.B., Leone T.C., Koves T., Gardell S.J., Krüger M., Hoppel C.L. (2016). The Failing Heart Relies on Ketone Bodies as a Fuel. Circulation.

[35] Bedi K.C., Snyder N.W., Brandimarto J., Aziz M., Mesaros C., Worth A.J., Wang L.L., Javaheri A., Blair I.A., Margulies K.B. (2016). Evidence for Intramyocardial Disruption of Lipid Metabolism and Increased Myocardial Ketone Utilization in Advanced Human Heart Failure. Circulation.

[36] Octavia Y., Tocchetti C.G., Gabrielson K.L., Janssens S., Crijns H.J., Moens A.L. (2012). Doxorubicin-induced cardiomyopathy: From molecular mechanisms to therapeutic strategies. J. Mol. Cel Cardiol..

[37] Raj S., Franco V.I., Lipshultz S.E. (2014). Anthracycline-Induced Cardiotoxicity: A Review of Pathophysiology Diagnosis, and Treatment. Curr. Treat. Options Cardiovasc. Med..

[38] Arai M., Yoguchi A., Takizawa T., Yokoyama T., Kanda T., Kurabayashi M., Nagai R. (2000). Mechanism of Doxorubicin-Induced Inhibition of Sarcoplasmic Reticulum Ca^2+^ -ATPase Gene Transcription. Circ. Res..

[39] Hayward R., Hydock D.S. (2007). Doxorubicin cardiotoxicity in the rat: An in vivo characterization. J. Am. Assoc. Lab. Anim. Sci..

[40] Bárdi E., Bobok I., Oláh A.V., Kappelmayer J., Kiss C. (2007). Anthracycline antibiotics induce acute renal tubular toxicity in children with cancer. Pathol. Oncol. Res..

[41] Kalender Y., Yel M., Kalender S. (2005). Doxorubicin hepatotoxicity and hepatic free radical metabolism in rats. Toxicology.

[42] El-Sayyad H., Ismail M., Shalaby F.M., Abou-El-Magd R.F., Gaur R.L., Fernando A., Raj M.H.G., Ouhtit A. (2009). Histopathological effects of cisplatin, doxorubicin and 5-flurouracil (5-FU) on the liver of male albino rats. Int. J. Biol. Sci..

[43] Storm G., Hoesel Q.G.C.M., van Groot G., de Kop W., Steerenberg P.A., Hillen F.C. (1989). A comparative study on the antitumor effect cardiotoxicity and nephrotoxicity of doxorubicin given as a bolus, continuous infusion or entrapped in liposomes in the Lou/M Wsl rat. Cancer Chemothe. Pharmacol..

[44] Yuan H., Zhang Q., Guo J., Zhang T., Zhao J., Li J., White A., Carmichael P.L., Westmoreland C., Peng S. (2016). A PGC-1*α*-Mediated Transcriptional Network Maintains Mitochondrial Redox and Bioenergetic Homeostasis against Doxorubicin-Induced Toxicity in Human Cardiomyocytes: Implementation of TT21C. Toxicol. Sci..

[45] Zhang S., Liu X., Bawa-Khalfe T., Lu L.-S., Lyu Y.L., Liu L.F., Yeh E.T.H. (2012). Identification of the molecular basis of doxorubicin-induced cardiotoxicity. Nat. Med..

[46] Luers C., Wachter R., Kleta S., Uhlir M., Koschack J., Scherer M., Binder L., Herrmann-Lingen C., Zapf A., Kulle B. (2010). Natriuretic peptides in the detection of preclinical diastolic or systolic dysfunction. Clin. Res. Cardiol..

[47] Ogawa T., Linz W., Stevenson M., Bruneau B.G., de Bold M.L.K., Chen J.H., Eid H., Schölkens B.A., de Bold A.J. (1996). Evidence for Load-Dependent and Load-Independent Determinants of Cardiac Natriuretic Peptide Production. Circulation.

[48] Hallows K.R., Mount P.F., Pastor-Soler N.M., Power D.A. (2010). Role of the energy sensor AMP-activated protein kinase in renal physiology and disease. Am. J. Physiol.-Renal. Physiol..

[49] Yin X.-N., Wang J., Cui L.-F., Fan W.-X. (2018). Enhanced glycolysis in the process of renal fibrosis aggravated the development of chronic kidney disease. Eur. Rev. Med. Pharmacol. Sci..

[50] de Boer I.H. (2008). Vitamin D and glucose metabolism in chronic kidney disease. Curr. Opin. Nephrol. Hypertens..

[51] Li A., Zhang W., Zhang L., Liu Y., Li K., Du G., Qin X. (2020). Elucidating the time-dependent changes in the urinary metabolome under doxorubicin-induced nephrotoxicity. Toxicol. Lett..

[52] Kagawa T., Zárybnický T., Omi T., Shirai Y., Toyokuni S., Oda S., Yokoi T. (2019). A scrutiny of circulating microRNA biomarkers for drug-induced tubular and glomerular injury in rats. Toxicology.

[53] Henneberry H.P., Aherne G.W. (1992). Visualisation of doxorubicin in human and animal tissues and in cell cultures by immunogold-silver staining. Br. J. Cancer.

[54] Szalay C.I., Erdélyi K., Kökény G., Lajtár E., Godó M., Révész C., Kaucsár T., Kiss N., Sárközy M., Csont T. (2015). Oxidative/Nitrative Stress and Inflammation Drive Progression of Doxorubicin-Induced Renal Fibrosis in Rats as Revealed by Comparing a Normal and a Fibrosis-Resistant Rat Strain. PLoS ONE.

[55] Brenner B.M. (1983). Hemodynamically mediated glomerular injury and the progressive nature of kidney disease. Kidney Int..

[56] Correa-Rotter R., Hostetter T.H., Manivel J.C., Rosenberg M.E. (1992). Renin expression in renal ablation. Hypertension.

[57] Hanifa M.A., Skott M., Maltesen R.G., Rasmussen B.S., Nielsen S., Frøkiær J., Ring T., Wimmer R. (2019). Tissue urine and blood metabolite signatures of chronic kidney disease in the 5/6 nephrectomy rat model. Metabolomics.

[58] Yang C.C., Chen Y.T., Chen C.H., Li Y.C., Shao P.L., Huang T.H., Chen Y.L., Sun C.K., Yip H.K. (2019). The therapeutic impact of entresto on protecting against cardiorenal syndrome-associated renal damage in rats on high protein diet. Biomed. Pharmacother..

[59] Faleiro L., Kobayashi R., Fearnhead H., Lazebnik Y. (1997). Multiple species of CPP32 and Mch2 are the major active caspases present in apoptotic cells. EMBO J..

[60] Liu H.-Z., Gao C.-Y., Wang X.-Q., Fu H.-X., Yang H.-H., Wang X.-P., Liu Y.-H., Li M.-W., Niu Z.-M., Dai G.-Y. (2012). Angiotensin(1-7) attenuates left ventricular dysfunction and myocardial apoptosis on rat model of adriamycin-induced dilated cardiomyopathy. Zhonghua Xin Xue Guan Bing Za Zhi.

[61] Gao J., Wei L., Song J., Jiang H., Gao Y., Wu X., Xu S. (2018). In vitro and in vivo study of the expression of the Syk/Ras/c-Fos pathway in chronic glomerulonephritis. Mol. Med. Rep..

[62] Ranjan A., Iwakuma T. (2018). Emerging Non-Canonical Functions and Regulation of p53. Int. J. Mol. Sci..

[63] Medeiros-Lima D.J.M., Carvalho J.J., Tibirica E., Borges J.P., Matsuura C. (2019). Time course of cardiomyopathy induced by doxorubicin in rats. Pharmacol. Rep..

[64] Armutcu F., Demircan K., Yildirim U., Namuslu M., Yagmurca M., Celik H.T. (2019). Hypoxia causes important changes of extracellular matrix biomarkers and ADAMTS proteinases in the adriamycin-induced renal fibrosis model. Nephrology.

[65] Kliem V., Johnson R.J., Alpers C.E., Yoshimura A., Couser W.G., Koch K.M., Floege J. (1996). Mechanisms involved in the pathogenesis of tubulointerstitial fibrosis in 5/6-nephrectomized rats. Kidney Int..

[66] Liu S., Kompa A.R., Kumfu S., Nishijima F., Kelly D.J., Krum H., Wang B.H. (2013). Subtotal nephrectomy accelerates pathological cardiac remodeling post-myocardial infarction: Implications for cardiorenal syndrome. Int. J. Cardiol..

[67] Yamada K., Endo S., Fukuda H., Abe Y., Yoshioka S., Itoh M., Kubota K., Hatazawa J., Satoh T., Matsuzawa T. (1985). Experimental studies on myocardial glucose metabolism of rats with 18F-2-fluoro-2-deoxy-d-glucose. Eur. J. Pediatr..

[68] Chaudhari U., Ellis J.K., Wagh V., Nemade H., Hescheler J., Keun H.C., Sachinidis A. (2017). Metabolite signatures of doxorubicin induced toxicity in human induced pluripotent stem cell-derived cardiomyocytes. Amino. Acids.

[69] Kobayashi M., Shikano N., Nishii R., Kiyono Y., Araki H., Nishi K., Oh M., Okudaira H., Ogura M., Yoshimoto M. (2010). Comparison of the transcellular transport of FDG and D-glucose by the kidney epithelial cell line, LLC-PK1. Nucl. Med. Commun..

[70] Kriz W., Napiwotzky P., Karger S.A.G. (1979). Structural and Functional Aspects of the Renal Interstitium1. Contributions to Nephrology.

[71] Rodrigues R.S., Bozza F.A., Hanrahan C.J., Wang L.-M., Wu Q., Hoffman J.M., Zimmerman G.A., Morton K.A. (2017). 18 F-fluoro-2-deoxyglucose PET informs neutrophil accumulation and activation in lipopolysaccharide-induced acute lung injury. Nucl. Med. Biol..

[72] Gounden V., Bhatt H., Jialal I. (2020). Renal Function Tests.

[73] Kety S.S. (1951). The theory and applications of the exchange of inert gas at the lungs and tissues. Pharmacol. Rev..

[74] Chade A.R. (2013). Renal vascular structure and rarefaction. Compr. Physiol..

[75] Potter D., Jarrah A., Sakai T., Harrah J., Holliday M.A. (1969). Character of Function and Size in Kidney During Normal Growth of Rats. Pediatric. Res..

