# Supplementary material for: 18F-FDG PET/MRI Imaging in a Preclinical Rat Model of Cardiorenal Syndrome—An Exploratory Study
Source: Int J Mol Sci. 2022 Dec 6;23(23):15409. doi: 10.3390/ijms232315409 (PMC9738874; doi:10.3390/ijms232315409)
Supplement: Supplementary file 1 [file ijms-23-15409-s001.zip › ijms-2059872-supplementary.pdf]

**Supplementary material**

# $^{18}$F-FDG PET/MRI Imaging in a Preclinical Rat Model of Cardiorenal Syndrome – An Exploratory Study

Dan M. Furcea[1,2,#], Laurențiu Agrigoroaie[1,2,#], Cosmin-T. Mihai[2], Ioannis Gardikiotis[2], Gianina Dodi[2*], Gabriela D. Stanciu[2], Carmen Solcan[3], Sorin I. Beschea Chiriac[3], Mihai M. Guțu[4], Cipriana Ștefănescu[4]

[1]Department of Nuclear Medicine, Sf. Spiridon University Emergency Hospital, Iași, Romania
[2]Advanced Research and Development Center for Experimental Medicine, Grigore T. Popa University of Medicine and Pharmacy of Iași, Romania
[3]Faculty of Veterinary Medicine, Ion Ionescu de la Brad University of Agricultural Sciences and Veterinary Medicine, Iași, Romania
[4]Department of Biophysics and Medical Physics - Nuclear Medicine, Grigore T. Popa University of Medicine and Pharmacy of Iași, Romania
[#]Equal contribution
*Corresponding author: Gianina Dodi

## 2.Materials and Methods

### 2.1 PET data extraction and analysis

PET data analysis was conducted by two independent evaluators, in two separate data extraction software environments: Carimas (Turku PET Centre©, Turku, Finland) and Vivoquant™ (InviCRO©, London, UK). As a first step of segmentation the PET and MR images were rescaled and merged. Averaged dynamic PET and MR images were reviewed for proper co-registration by manual adjustments on a region-by-region basis. More details can be found in Supplementary material.

Volumes of interest (VOI) were drawn according to Figure S1:

- heart (noted as 1) was sampled in two regions of the left ventricle, intracavitary and ventricular free wall avoiding apical region;
- vascular VOIs (noted as 8), one for aorta and one for inferior vena cava (superior to the renal pedicle) were obtained from dynamic merged images, when the FDG bolus was present there;
- cortical (7), medullary (4) and renal pelvis VOIs (3) were placed by selecting physiologically relevant time frames;
- liver VOIs (6) were obtained with a spherical shape inside the left hepatic lobe;
- skeletal muscle VOIs (2), of cylindrical shape, were sampled inside of the psoas muscle.

Data were extracted from the VOIs as the mean activity, at each time-point.

Dynamic reconstructed studies were analyzed using several approaches. SUV normalized data points were pre-processed by searching and applying a proper smoothing algorithm for denoising purposes. It was noticed that a low-pass parabolic Fourier filter followed by a Lowess would suffice, both with a narrow sampling bandwidth as to minimize

curve gradient distortions. For compartmental analysis, image-derived input function (IDIF) from inferior cava VOIs was chosen as suggested by Lanz et al. [24] and Huang et al. [25].

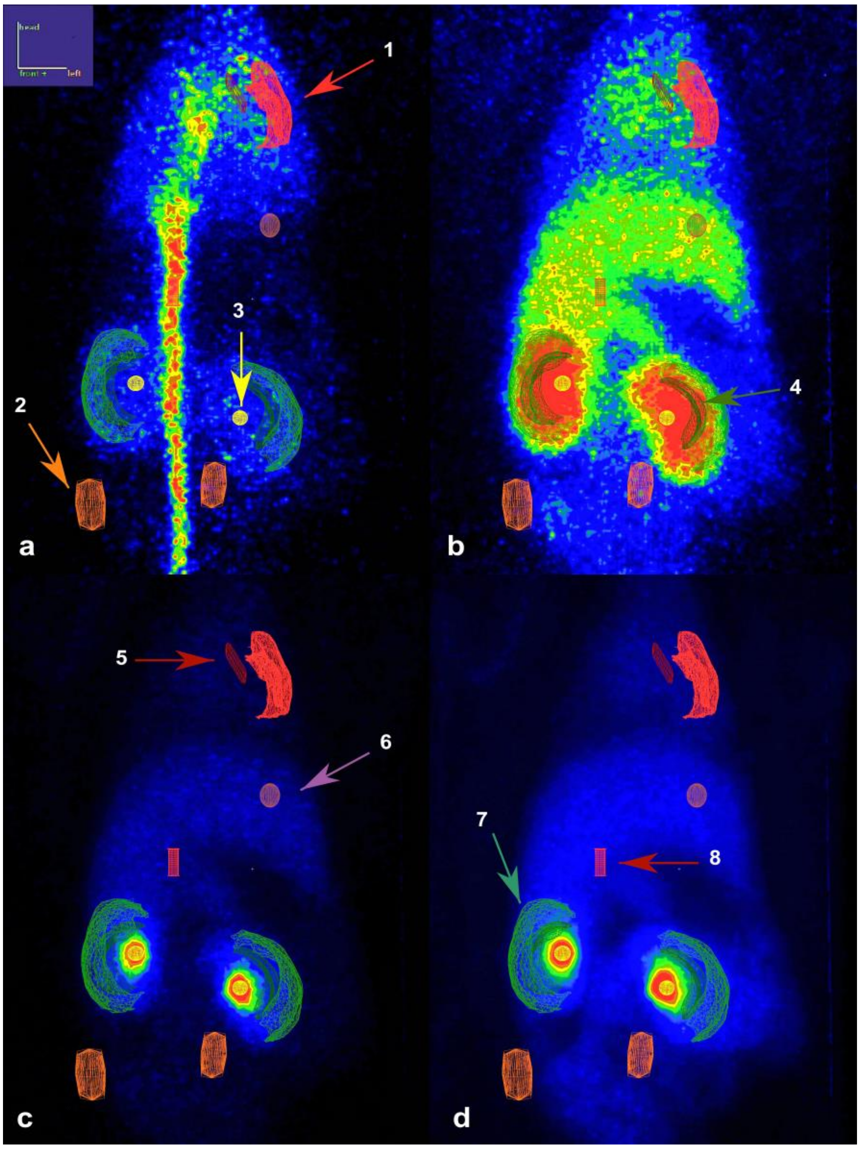


**Figure S1.** $^{18}$F-FDG PET of a control group rat showing, in maximal intensity projection display, the location of each drawn VOI, manually sculpted during relevant time frames for each organ (a- vascular distribution after injection; b- organ perfusion phase; c- renal excretion phase; d- steady-state equilibrium phase); numbers embedded and explained in text (1- heart (left ventricular VOI); 2- skeletal muscle VOI; 3- renal pelvis VOI; 4- renal medullary VOI; 5- Intraventricular VOI. 6- liver VOI; 7- renal cortical VOI; 8- vascular VOI)

The first 5 minutes of the dynamic study were used for compartmental analysis of PET data. A three compartments model with trapping (i.e. k4 fixed to 0), standard for $^{18}$F-FDG studies [26], with blood bolus delay correction and tissue blood fraction simultaneous estimation was fit to left ventricle, liver, skeletal muscle, renal cortical and medullary regions. The system of coupled ordinary differential equations describing tracer dynamics was solved by a relative weighted non-linear least squares Levenberg-Marquadt algorithm with linear

least squares initialization and 30 random initializations for a better choice of initial parameter values, as below:

$$C_{model}(t) = \begin{cases} \frac{dC_e(t)}{dt} = K_1 C_{IDIF}(t) - (k_2 + k_3)C_e(t) \\ \frac{dC_m(t)}{dt} = k_3 C_e(t) \end{cases}$$

where $C_{model}(t)$, $C_e(t)$, $C_m(t)$ and $C_{IDIF}(t)$ are predicted tissue, extravascular, metabolized and image-derived input function tracer concentration at time t, while $K_1$ (mL/g/min), $k_2$ (1/min) and $k_3$ (1/min) are model estimated microparameters for blood-to-tissue, tissue-to-blood transfer rates and intracellular phosphorylation rate.

Uncertainty in estimation of model microparameters was observed by employing a Monte Carlo bootstrapping of the weighted residuals [27] with resampling from measured data (n=400 simulations). Net uptake (Ki) and glucose metabolic rates (MRGlu) were calculated according to:

$$K_i = \frac{K_1 k_3}{k_2 + k_3} \qquad MRGlu = K_i \times \frac{PG}{LC}$$

where $K_i$ stands for net influx rate, PG is glucose plasma concentration (mmol/L) and LC is the lumped constant which relates differences in intracellular transport and phosphorylation between $^{18}$F-FDG and glucose, in this study fixed to 1 as it was not determined experimentally [28].

The data was further processed with the goal of performing an early Patlak curve analysis (for the vascular time, and renal excretion), using the various kidney sampled regions and the vascular compartment as input function. The most reliable and consistent input function was the one derived from the Aorta for this purpose, as it was fairly readily identifiable and had a very similar plot through the studied individuals [28]. The data was derived by plotting $\frac{R(t)}{Cp(t)}$ against $\frac{\int_0^t Cp(\tau)d\tau}{Cp(t)}$, where *R(t)* is the activity in the region of interest and Cp(t) would be the plasma concentration of tracer, but in our case we used an image-derived input function from the Aorta.

Given the one-second reconstruction duration, the plotted data was inherently noisy. As a first step, we aimed at determining the best smoothing algorithm, which will maintain the curve characteristics, like the slopes, peak location, and general point amplitude. Various methods were tested, as Savitzky-Golay, Percentile filter, Fast Fourier transform filter, finally selecting Adjacent Averaging with 30 points of window as the most suitable one. All curves were smoothed once, in the beginning, using this algorithm.

Often the Aorta is not readily identifiable, even on fused PET/MRI images. In this case, we start each PET acquisition with a few seconds delay prior to tracer administration. This time gap, and the short 1s reconstructed images, gave us the frames necessary for clear vessel identification. Thus, the Aorta was sampled for each individual, in the corresponding region. Therefore, the input function could be used in the form of integral of aorta activity over time divided by aorta activity at each time point (IntAo/Ao) as the independent variable of the Patlak plot.

Four main regions were identified and sampled using the post administration time dependent vascular and tissue tracer location. Thus, the cortex, medulla and pelvic region of the kidney and liver were identified. To obtain the dependent axis values, the activity curves from these regions were divided by the aorta activity. The resulting values for the dependent and independent axis were plotted against each other, thus resulting the Patlak plot.

As a structural parameter, derived from MRI sequences, an attempt was made at measuring potentially relevant dimensions of target organs. Since there was no respiratory or cardiac gating of MRI signal, renal volumetric measurements were too noisy to rely on, therefore, estimations based on length in axial plane and thickness of antero-posterior and lateral border to papilla in trans axial plane were generated. Also, an ellipsoid approximation was used for kidney volume estimation.

## 3. Results

### 3.1. Mortality and study design

Following the guidelines developed by Shen et al. [20] and Zhang & Kompa [21] in modeling dilated cardiac failure and chronic kidney disease, at day 0, we formed a pooled L2+L3 doxorubicin-induced heart failure group and also an L4 progressive kidney failure group. Until the point in time of L3 and L4 entry (Figure 2), the survival curve was uneventful for all lots under study.

In the case of L3 rats, in the first week after subtotal nephrectomy, we registered a steep decline in survival with complete eradication of group members by the time of scheduled PET scan. In contrast, L4 individuals seemed to tolerate the doxorubicin regimen for about 4-5 weeks, when survival began to drop, but with a mortality of about 70%.

In this setting, the L3 group was supplemented from the L2+L3 pool, treated only with 6 weeks doxorubicin, and examined them as a late versus early model of dilated heart failure, potentially accompanied by renal impairment. Hence, at the time of $^{18}$F-FDG PET scans, group L3 consisted of 4 rats, while L4 had left only 3 individuals.

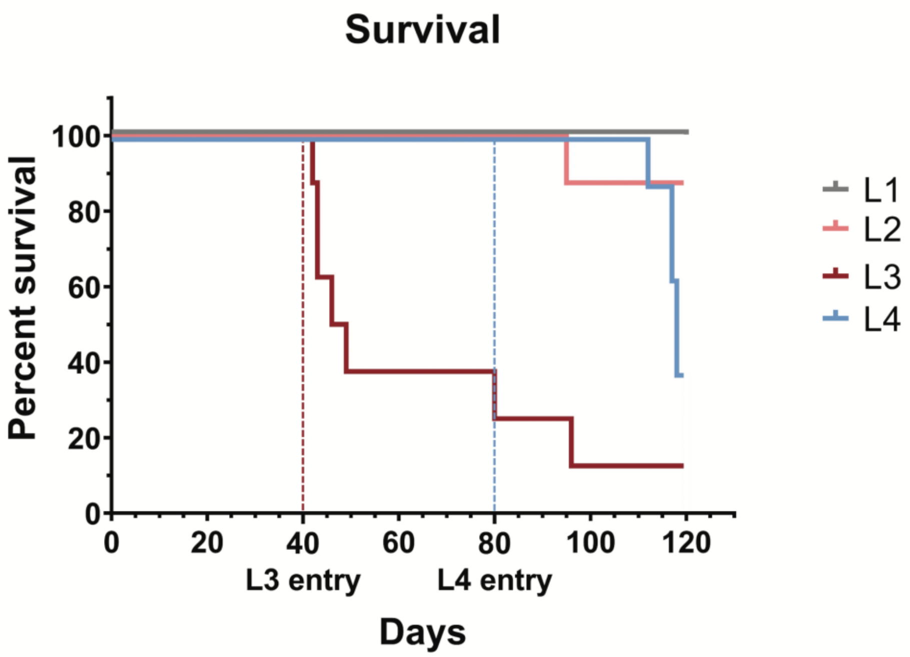


**Figure S2.** Kaplan-Meier curve depicting a faster decline in survival for cardiorenal group in comparison to renocardiac rats.

## 4. Discussion

From the perspective of model feasibility, during the course of our study, we observed that doxorubicin-pretreated rats followed by acute renal failure induced by 5/6

nephrectomy results in unexpectedly high mortality rates. Since timing of interventions and time elapsed since doxorubicin initiation can play a major role, an earlier renal intervention or a 3/6 nephrectomy might allow for a wider survival window and a valid working model for cardiorenal syndrome. The reverse situation, as an analogue of renocardiac syndrome, where initiation is done by 5/6 nephrectomy and, after development of chronic renal failure, doxorubicin is started, is somewhat better tolerated, but also with an unacceptable rate of mortality for effective examination of target phenomena.

References

20. Shen L.-J., Lu S., Zhou Y.-H., Li L., Xing Q., Xu Y.-L. Developing a rat model of dilated cardiomyopathy with improved survival. *J Zhejiang Univ-Sci B* **2016**, *17(12)*, 975–983.

21. Zhang Y., Kompa A.R. A practical guide to subtotal nephrectomy in the rat with subsequent methodology for assessing renal and cardiac function. *Nephrology* **2014**, *19(9)*, 552–561.

24. Lanz B., Poitry-Yamate C., Gruetter R. Image-Derived Input Function from the Vena Cava for 18F-FDG PET Studies in Rats and Mice. J Nucl Med 2014, 55(8), 1380–1388.

25. Huang Q., Massey J.C., Mińczuk K., Li J., Kundu B.K. Non-invasive determination of blood input function to compute rate of myocardial glucose uptake from dynamic FDG PET images of rat heart in vivo: comparative study between the inferior vena cava and the left ventricular blood pool with spill over and partial volume corrections. Phys Med Biol 2019, 64(16), 165010.

26. Bertoldo A., Vicini P., Sambuceti G., Lammertsma A.A., Parodi O., Cobelli C. Evaluation of compartmental and spectral anal-ysis models of [18/F]FDG kinetics for heart and brain studies with PET. IEEE Trans Biomed Eng 1998, 45(12), 1429–1448.

27. Kukreja S.L., Gunn R.N. Bootstrapped DEPICT for error estimation in PET functional imaging. NeuroImage 2004, 21(3), 1096–1104.

28. Doenst T. Complexities Underlying the Quantitative Determination of Myocardial Glucose Uptake with 2-Deoxyglucose. J Mol Cell Cardiol 1998, 30(8),1595–1604.
